# Taxonomic identity of four groups of *Glandirana rugosa* (Anura, Ranidae) in Japan revealed by the complete mitochondrial genome sequence analysis

**DOI:** 10.1080/23802359.2020.1833772

**Published:** 2020-11-11

**Authors:** Masatake Mochizuki, Yoriko Nakamura, Masahisa Nakamura

**Affiliations:** a Graduate School of Advanced Science and Engineering, Waseda University, Tokyo, Japan; b Department of Science Education, Ehime University, Matsuyama, Japan; c Waseda Research Institute for Science and Engineering, Waseda University, Tokyo, Japan

**Keywords:** Amphibian mtDNA, mitogenome, phylogenetic tree, *Glandirana rugosa*, new species

## Abstract

The Japanese *Glandirana rugosa* phylogenetically consists of four groups. However, the taxonomic identity of these groups still remains unclear. We determined the complete mitogenome sequences of the four groups of *G. rugosa*. The mitogenomes were 17,394–17,781 bp in length. The phylogenetic analysis clearly showed that the genus *Glandirana* is monophyletic and that the four groups of *G. rugosa* are separated into two clusters: one cluster represents *G. rugosa*, the other cluster may represent a different species.

The Japanese *Glandirana rugosa* was one of the five frog species collected by Siebold during his stay in Nagasaki (Temminck and Schlegel 1838). Originally, this frog was described as *Rana rugosa* Schlegel in Temminck and Schlegel (1838). Later, the species was transferred into the genus *Glandirana* (Frost 2013). The *Glandirana rugosa* is now separated into four groups, designated as the West, East, North, and Central groups, based on the mitochondrial *12S rRNA* gene (Oike et al. 2017). This suggests that the Japanese *G. rugosa* probably consists of cryptic species. We collected individuals of *G. rugosa* in the Cities of Higashi-Hiroshima (West; 34°29′03.6″N 132°42′13.4″E), Nagaoka (North; 37°21′32.7″N 138°55′26.1″E), Shizuoka (Central; 35°06′28.1″N 138°21′51.2″E), and Kamogawa (East; 35°08′22.8″N 140°12′32.2″E). We stored the tissues in Dr. Nakamura lab, Ehime University (sample database number: NSE_A00101-4). DNA was extracted (mtDNA extractor CT kit, Wako, Osaka, Japan) and cleaved with *Pst*I and *EcoR*I which yielded suitable DNA size fragments for cloning. Cloned DNA fragments were Sanger sequenced (following Sumida et al. 2001) and the mitogenome of *Pelophylax nigromaculatus* (GenBank ACCN AB043889) was used as reference for assembling. The complete mitogenomes of the four groups of *G. rugosa* (GenBank ACCN LC536281-4) were 17,394–17,781 bp in length, consisting of 13 protein-coding genes, 12S and 16S rRNAs, 21 tRNAs, and one pseudo-tRNA and one non-coding D-loop. The RAxML (Figure 1) and MrBayes phylogenetic trees revealed the monophyly of the genus *Glandirana* in which *G. rugosa*-West and -North, and *G. emeljanovi* were separated from *G. rugosa*-East and -Central (Figure 1); however, the latter tree is not shown because of a figure limit. The Korean *G. emeljanovi* is probably a different species from the Japanese *G. rugosa*, based on the relatively low genetic identity between *G. emeljanovi* and *G. rugosa* (probably West group) mitogenomes (84.0%) given the range of intraspecific genetic identity in Ranidae frogs is 94.0–100.0% (Eo et al. 2019). In this study, we found that genetic identity values of mitogenomes of *G. rugosa*-North, -East, -Central, and *G*. *emeljanovi* were 88.6, 81.8, 82.4, and 84.8%, respectively, when the *G. rugosa*-West was set as 100%. We also show homology of nucleotide sequences of 13 protein-coding genes among the four groups in Table S1. From these results, we concluded that *G. rugosa*-West and -North are a different species from *G. rugosa*-East and -Central, and from the Korean *G*. *emeljanovi*. Thus, the Japanese *G. rugosa* is separated into at least two independent species. The West group likely represents *G. rugosa* s.str., because it contains individuals from the supposed type locality, Nagasaki (Oike et al. 2017). We argue that the East and Central groups should have a new species name.

**Figure 1.**
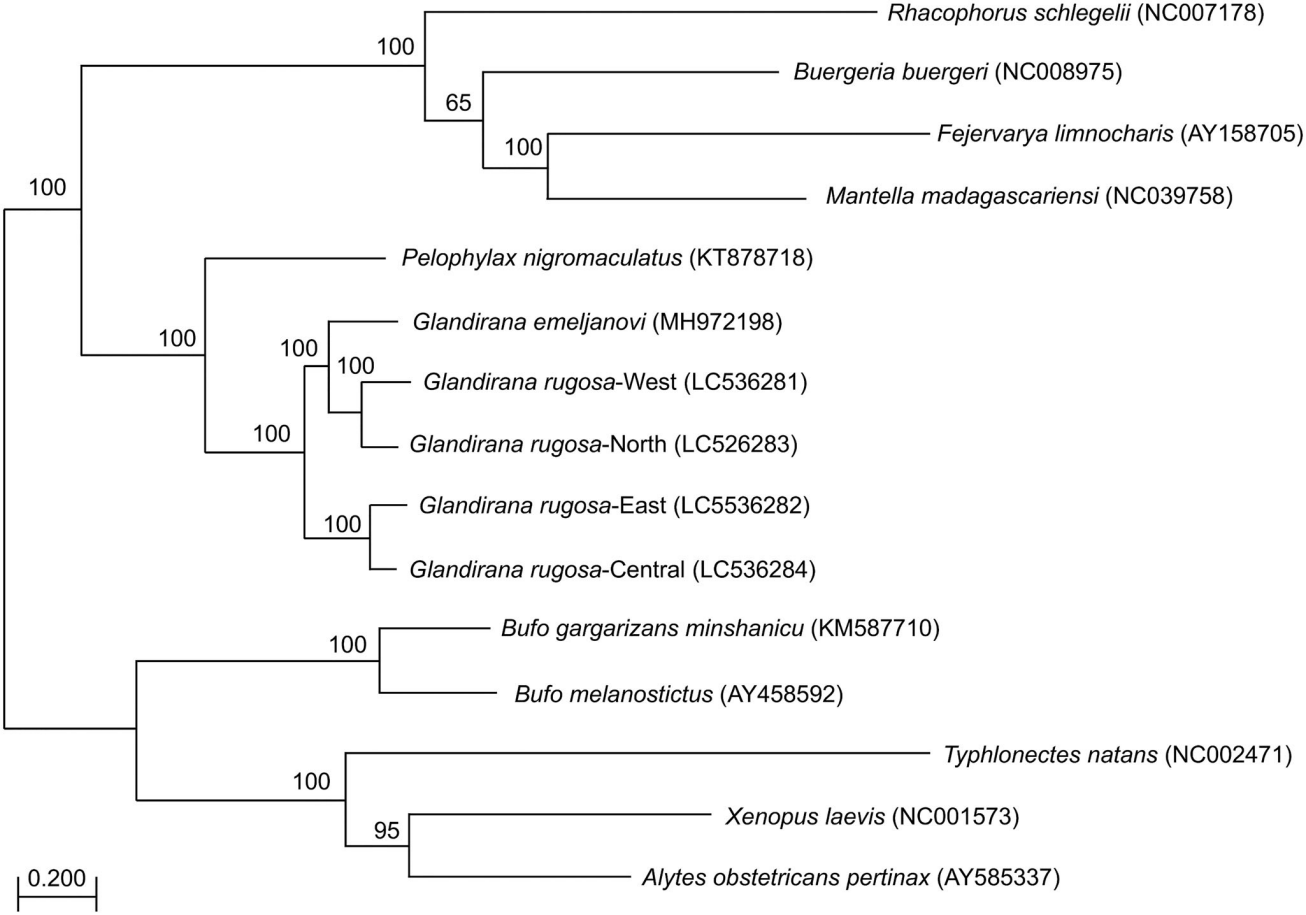
RAxML tree based on whole mitogenomes from different species. Numbers at each node indicate the bootstrap values of 1000 replications from the tree. GenBank ACCN is shown in parentheses.

## Data Availability

The data are available at the website (https://www.ncbi.nlm.nih.gov/genbank/) under ACCN LC536281-4.
